# Retraction Note: *Gemella massiliensis* sp. nov., a new bacterium isolated from the human sputum

**DOI:** 10.1007/s00203-024-04147-5

**Published:** 2024-09-26

**Authors:** Maxime Descartes Mbogning Fonkou, Cheikh Ibrahima Lo, Zouina Mekhalif, Melhem Bilen, Enora Tomei, Edmond Kuete Yimagou, Grégory Dubourg, Didier Raoult, Florence Fenollar, Pierre-Edouard Fournier

**Affiliations:** 1 Aix Marseille Univ, IRD, AP-HM, MEPHI, Marseille, France; 2 Institut Hospitalo-Universitaire Méditerranée-Infection, 19-21 Boulevard Jean Moulin, 13385 Marseille Cedex 05, France; 3 Aix Marseille Univ, IRD, AP-HM, SSA, VITROME, Marseille, France; 4 Special Infectious Agents Unit, King Fahd Medical Research Center, King Abdulaziz University, Jeddah, Saudi Arabia

**Retraction Note: Archives of Microbiology (2021) 203:5817–5823**

10.1007/s00203-021-02493-2

The Editor has retracted this paper because the authors could not provide evidence of approval from an appropriate ethics committee.

The authors did not respond to correspondence from the Publisher about this retraction.

